# Mosquito control practices and perceptions: An analysis of economic stakeholders during the Zika epidemic in Belize, Central America

**DOI:** 10.1371/journal.pone.0201075

**Published:** 2018-07-19

**Authors:** Molly Duman-Scheel, Kathleen K. Eggleson, Nicole L. Achee, John P. Grieco, Limb K. Hapairai

**Affiliations:** 1 Department of Medical and Molecular Genetics, Indiana University School of Medicine, South Bend, Indiana, United States of America; 2 Eck Institute for Global Health, University of Notre Dame, Notre Dame, Indiana, United States of America; 3 Department of Biological Sciences, University of Notre Dame, Notre Dame, Indiana, United States of America; 4 Department of Medicine, Indiana University School of Medicine, South Bend, Indiana, United States of America; University of Miami, UNITED STATES

## Abstract

The tourist-based economy of Belize, a tropical hub for eco-tourism, is at high risk to be disproportionately impacted by established and emerging mosquito-borne diseases such as Zika. An online survey was used to probe economic stakeholders working in the Belize tourism industry about their mosquito control practices and perceptions. Responses demonstrated that the respondents have good working knowledge of mosquitoes and mosquito-borne illnesses. Most businesses surveyed engage in some means of mosquito control, either through larval source reduction or use of insecticides on the premises. Larvicide use was significantly correlated with a general willingness to use insecticides, as well as belief that treatment of water will reduce mosquito densities and disease transmission. A majority of the respondents agreed that they would be interested in buying a new larvicide to be used on the business premises if it were shown to be safe and effective. The safety of mosquito control products for humans, animals, plants, and the environment in general, followed by product effectiveness, are the most critical determinants of mosquito control purchasing decisions. A majority of respondents agreed that control of mosquitoes and mosquito-borne illnesses is central to the success of their tourist-based industry. Respondents expressed significant concern that the Zika epidemic was over-sensationalized by the media, and that this negatively impacted their livelihoods. The respondents, many of whom are associated with eco/sustainable businesses, also voiced concerns that chemical pesticides could have a negative impact on human health and the environment and expressed a desire for balance between effective mosquito control and preservation of the rich biodiversity of Belize. This study provided a framework for further engagement activities in Belize and other Caribbean nations, uncovered both concerns and support for emerging mosquito control technologies, and revealed opportunities for further debate and educational outreach efforts.

## Introduction

Mosquito-borne illnesses such as dengue and Zika are spread primarily through the bite of infected female *Aedes* mosquitoes. Dengue is one of the most significant mosquito-borne illnesses in the tropics and subtropics. More than a third of the world’s population is at risk for contracting dengue virus, and as many as 400 million people are infected annually. Belize has an ongoing risk of dengue transmission, with 2,958 cases of dengue being confirmed in Belize in 2017 [1], and dengue is a leading cause of febrile illness among travelers returning from the Carribean [2]. Zika was designated a public health emergency of international concern in 2016. In addition to being transmitted by mosquitoes, Zika virus can be transmitted sexually, and it can be passed from a pregnant woman to her fetus. Fetal infection with Zika virus can result in severe birth defects, including microcephaly. Cases of Zika, which have also been linked to Guillain-Barré syndrome, a serious neurological disorder, are currently occurring in many countries in the Americas, including Belize [3, 4].

In 2016, a U.S. traveler returning from Belize reported an imported case of Zika virus in epidemiological week 14 (EW 14) [5]. In 2017, the number of reported/suspected cases of Zika peaked during EWs 7 and 8, with ~165 cases suspected and 45 cases confirmed. Although no reported cases of Guillain-Barré syndrome have yet been associated with Zika in Belize, two suspected cases of congenital syndromes associated with Zika have been reported by the Belize authorities [5]. Belize has both air and sea travel for tourists. Five cruise lines that offer tours to the islands and the mainland visit weekly [6], and beaches are frequented by tourists who can become infected with Zika virus and take it home to the United States, Europe, and other regions [7]. In April 2016, the CDC issued an alert Level 2 warning for travelers to Belize, indicating that pregnant women should not travel to Belize, an area with risk of Zika, and that partners of pregnant women and couples planning pregnancy should take preventive steps to avoid being infected [8]. There are presently no medicines to cure or human vaccines to prevent Zika, dengue, or most other mosquito-borne illnesses. Consequently, controlling mosquitoes is the primary means of preventing mosquito-borne diseases [9].

The Belize Ministry of Health (MOH) [10] oversees a large vector control program that aims to protect both the citizens of Belize and visitors to the country from mosquito-borne illnesses. Mosquito surveillance is conducted weekly using standard immature indices [11] to monitor need for control measures [12]. *Aedes* mosquitoes lay eggs in water-filled containers located within or close to human residences [13], and larviciding, the treatment of container breeding sites with chemical or microbial agents that kill *Aedes* larvae, is therefore a major component of integrated *Aedes* mosquito control and disease prevention programs [14]. Mosquito control measures employed by the Belize MoH include use of the larvicide Abate® to target immatures and ultra-low volume truck mounted spraying for adult control. Thermal fogging inside homes is used for index disease cases and the 30 homes surrounding the case house [12]. The Belize MoH also conducts monthly health fairs to help educate people about mosquito control and vector-borne illnesses [10]. Unfortunately, due to insecticide resistance and escalating concerns for the negative effects of pesticides on non-target species, mosquitoes are becoming increasingly difficult to control [9, 15]. New strategies for combating established and emerging arthropod-borne infectious diseases are vitally necessary in Belize and worldwide.

We recently developed interfering RNA larvicides that kill up to 100% of mosquito larvae in laboratory trials [16, 17]. We have identified several potential delivery systems for these larvicides [16, 17] and are evaluating their efficacy in semi-field trials. The input of intended users is crucial for the ultimate acceptability and practical efficacy of proposed interventions as they are being developed [18]. To this end, we have adapted an approach that epitomizes pursuit of Responsible Research and Innovation (RRI) [19], a transparent, interactive process by which societal actors and innovators become mutually responsive to each other with a view to the acceptability, sustainability, and societal desirability of the innovation process and its marketable products. As described by Lavery et al. [18], effective community engagement in global health studies serves to provide investigators an opportunity to ensure that the purpose and goals of the research are clear to the community, establish relationships and commitments to build trust with relevant community authorities, and allow researchers to understand the community, its diversity, changing needs, and assets. Engagement also serves to maximize opportunities for stewardship, ownership, and shared control by the community, provide a platform for expression of dissenting opinions or in extreme cases, prohibition of the research, and give the researchers an opportunity to modify the proposed research strategies, as needed.

Here, we report the findings from an internet-based assessment survey of economic stakeholders in the Belize tourism industry. The study had four primary aims: 1) to understand the importance of mosquito control to putative economic stakeholders in the Belize tourism industry, especially with respect to the recent Zika virus epidemic, 2) to assess, in general, current mosquito control practices of these stakeholders, 3) to evaluate current uses and perceptions of larviciding in Belize, and 4) to examine attitudes toward new mosquito control technologies, including new larvicidal biocontrol agents in a Zika endemic country. The results of this investigation provided insight into existing levels of mosquito knowledge and control efforts in Belize, as well as attitudes toward the Zika epidemic, larviciding, and the potential for new control measures. This study design provides a framework for further engagement activities in Belize and additional field sites and revealed opportunities for additional educational outreach efforts.

## Materials and methods

### Ethics statement

This study was approved by the Belize Ministry of Health and the Indiana University Office of Research Compliance (Study #1608074907).

### Survey administration

The survey (S1 File), including extension of invitations to participate and collection of informed consent, was administered online through the Qualtrics platform. Survey invitations were sent via email to 1,073 adults who held senior/executive positions in for-profit businesses associated largely or primarily with the tourism industry in Belize. The Belize Board of Tourism provided the list of relevant businesses and email addresses. Invitations were sent on March 29, 2017, and reminders were issued through April 26, 2017. The survey, which consisted of a combination of 40 five-point Likert scale items, fill-ins, and open-ended writing prompts intended to probe the concerns, current practices, and anticipated future needs of economic stakeholders with respect to mosquito control in general, and more specifically, regarding use of larvicides and new mosquito control technologies. Routine demographic data were electronically collected along with survey responses. No individually identifying information was collected. Although a Spanish translation of the survey was offered (S2 File), only one survey respondent opted to use it. His/her responses to open questions were translated into English for subsequent textual analyses.

### Data analysis

Results were filtered to exclude responses from individuals who did not complete the survey. Likert scale responses were subjected to statistical and factor analyses with Qualtrics Stats iQ software. Statistical comparisons were conducted between responses to each individual survey question (variable) and all other questions (variables) in the data set. All statistically significant results observed among these comparisons are reported and discussed herein. The Stats iQ software makes a recommendation as to the most appropriate statistical test for the data being analyzed; the statistical comparisons reported herein adhered to these recommendations. Moreover, the Stats iQ software program alerts users when the size of the data set is too small for reliable statistical comparisons. Importantly, no such alerts were noted for the analyses reported here.

Responses to open questions on the importance of mosquito control and the impact of Zika were coded as negative or positive and weighted 1–5 using the Likert scale, with strongly disagree corresponding to 1 and strongly agree corresponding to 5. Coded data were uploaded and statistically evaluated in conjunction with responses to the other survey questions using Qualtrics Stats iQ software. Word count analyses of all open-ended response questions were performed using TextAnalyzer [20], and the results of these analyses, in addition to inspection of the open-ended responses by the researchers, were used as a basis to further code positive and negative responses. Moreover, representative quotes were selected to illustrate the general sentiments found among each set of coded responses and are discussed herein. Analysis of the fourth open-ended response question, which asked responders to comment further on any areas of their choice, did not uncover any additional findings that were not identified through analysis of the rest of the open-ended responses or survey data, and was excluded from this report.

## Results and discussion

### Business and respondent demographics

**Fig 1:**
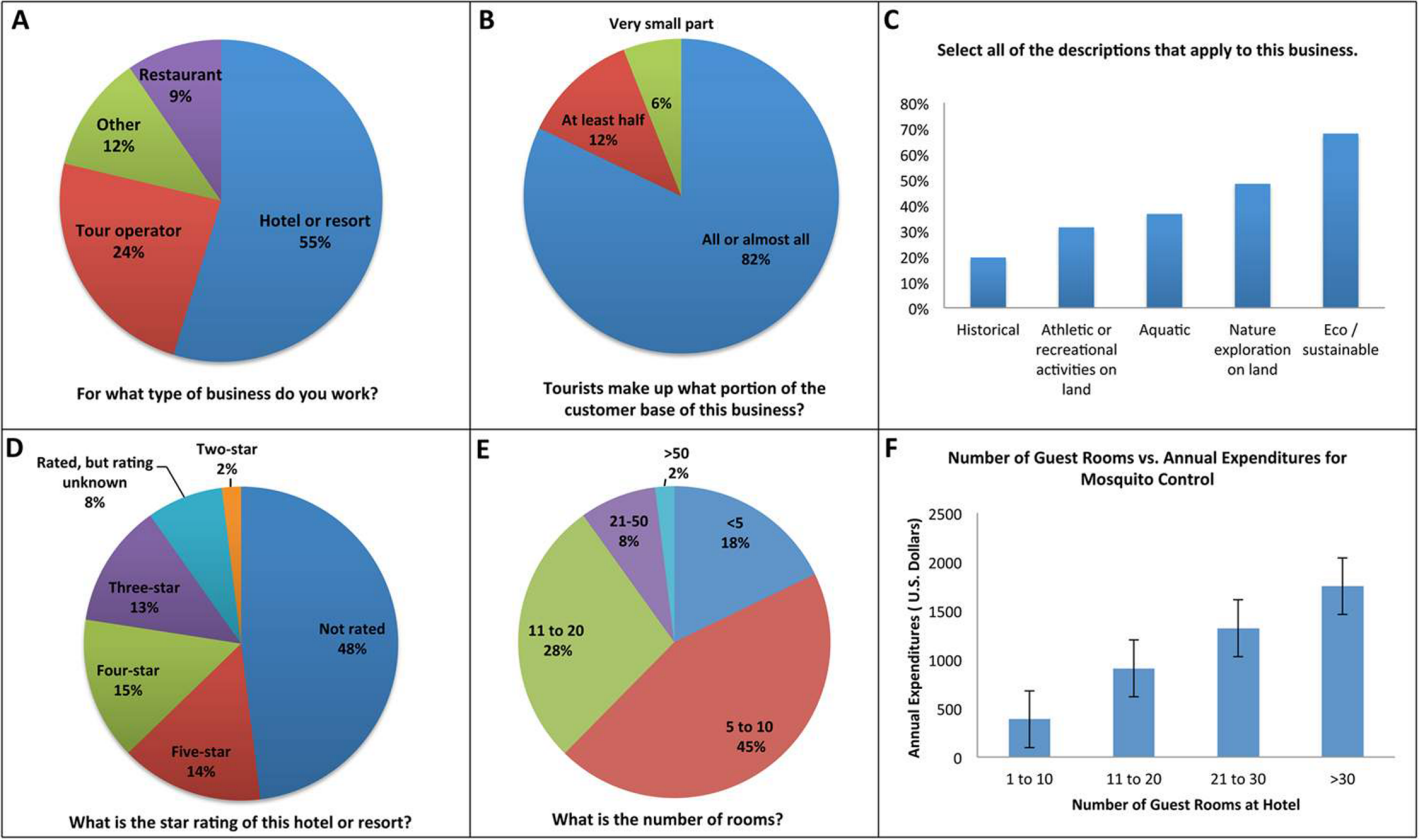
Summary of business demographic data. Summaries of respondent-supplied information regarding the businesses that they owned or in which they were employed are shown: A) Type of business, B) Proportion of business that is tourist-based, C) Description of business, D) Star rating, E) Number of guest rooms, F) Number of guest rooms vs. total annual U.S. dollars spent by the establishment for mosquito control. Percentages in A-E correspond to the percentage of the total respondents that provided the indicated answer (respondent count numbers are provided in Table 1).

**Fig 2:**
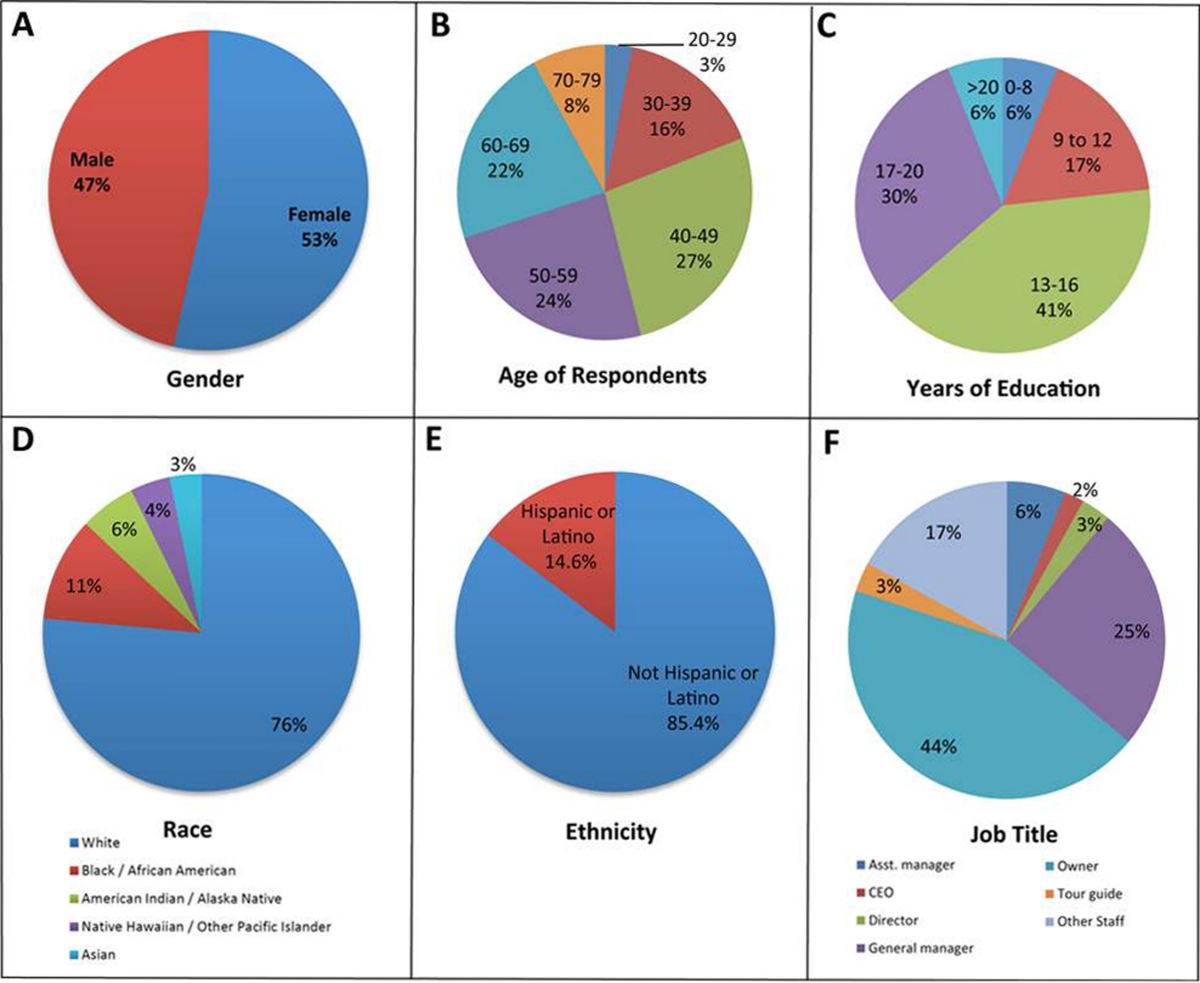
Demographics of the survey respondents. Self-reported demographic data provided by the respondents is shown: A) Gender, B) Age in years, C) Years of formal education, D) Race, E) Ethnicity (note that race and ethnicity categories correspond to those of the U.S. census), and F) Job title. Percentages in A-F correspond to the percentage of the total respondents that provided the indicated answer (respondent count numbers are provided in Table 2).

Subjects for this study consisted of adults who held senior/executive positions in for-profit businesses dependent in part or entirely upon Belize tourism for financial sustainability. 228 individuals of the 1,073 subjects invited initiated the survey, and 168 of these individuals completed the survey (16% completion rate, median completion time = 16 minutes). The businesses represented in this study included hotels and resorts, restaurants, and tour operators (Fig 1A and Table 1), and over 80% were supported primarily through a tourist-based customer base (Fig 1B and Table 1). A third of the businesses were eco/sustainable, and 57% were associated with athletic or recreational based activities that occurred both on land and in water (Fig 1B and Table 1). Over half of the respondents worked in hotels or resorts, the majority of which had a two to five star ranking (Fig 1D and Table 1), and 73% of which had 5–20 rooms for rent (Fig 1E and Table 1). Nearly 70% of the respondents were owners, general managers, or directors with management or executive duties (Fig 2F and Table 2). 81% of the respondents were over the age of 40 (Fig 2B and Table 2), and 76% of the respondents had 13 or more years of formal education (Fig 2C and Table 2). Just over half of the respondents were female (Fig 2A and Table 2), and >75% were white and non-Hispanic/Latino (Fig 2E and Table 2). Given that the target subject population was rather select, the small number of survey respondents is not unexpected, but may not be representative of all of Belize. Despite the small sample size, the proportion of respondents working for a particular business type correlated well with the proportion of invited subjects who worked for a particular type of business. For example, the percentage of respondents working in hotels and resorts (55%, Fig 1D and Table 1) corresponded to the proportion of invited subjects who worked for hotels and resorts (53% of the 1,073 total invitees). Thus, despite the small sample size, it is does not appear that respondents working for a particular business type were over- or under-represented in this study.

**Table 1:** Business demographics.

Question	Answer	% of Responses	Count
**For what type of business do you work?**	Hotel or resort	55%	103
	Restaurant	10%	18
	Tour operator	24%	45
	Other	12%	22
	Total	100%	188
**Select all of the descriptions that apply to** **the business.**	Eco / sustainable	33%	104
	Historical	10%	30
	Aquatic	18%	56
	Athletic or recreational activities on land	15%	48
	Nature exploration on land	24%	74
	Total	100%	312
**What is the star rating of this hotel or resort?**	One-star	0%	0
	Two-star	2%	2
	Three-star	13%	13
	Four-star	15%	15
	Five-star	15%	15
	Rated, but do not know	8%	8
	Not rated	48%	49
	Total	100%	102
**What is the number of rooms?**	<5	18%	18
	5 to 10	45%	45
	11 to 20	28%	28
	21–50	8%	8
	>50	2%	2
	Total	100%	101
	Mean = 8 rooms, C.I. = 9–13 rooms		
**Tourists, foreign or domestic, make up what portion of the customer base of this business?**	A very small part	6%	9
	At least half	12%	18
	All or almost all	82%	124
	Total	100%	151

Business Demographic Data. Related survey questions, as well as the percentages/counts of respondents with the indicated answers are shown. C.I. = Confidence interval.

**Table 2:** Respondent demographics.

Question	Answer	% of Responses	Count
**What is your gender?**	Male	47%	60
	Female	53%	69
	Total	100%	129
**What is your age?**	20–29	3%	4
	30–39	16%	20
	40–49	27%	35
	50–59	24%	31
	60–69	22%	29
	70–79	8%	10
	Total	100%	129
	Mean = 51 yrs., C.I. = 49–54 yrs.		
**Years of formal education completed?**	0–8 (Primary)	6%	8
	9 to 12 (Secondary)	17%	22
	13–16 (Tertiary)	40%	52
	17–20 (Advanced)	30%	39
	>20 (Advanced+)	6%	8
	Total	100%	129
	Mean = 15 yrs., C.I. = 14–16 yrs.		
**What is your race?**	Asian	3%	4
	American Indian / Alaska Native	6%	7
	Black / African American	11%	13
	Native Hawaiian / Other Pacific Islander	4%	5
	White	76%	94
	Total	100%	123
**What is your ethnicity?**	Not Hispanic or Latino	85%	105
	Hispanic or Latino	15%	18
	Total	100%	123
**What is your professional title?**	Asst. manager	6%	9
	CEO	2%	3
	Director	3%	5
	General manager/Director	25%	40
	Owner	44%	70
	Tour guide	3%	4
	Other Staff	17%	27
	Total	100%	158

Demographic questions, as well as the percentages/counts of respondents with the indicated answers are shown. C.I. = Confidence interval.

### Mosquito knowledge and control practices

**Fig 3:**
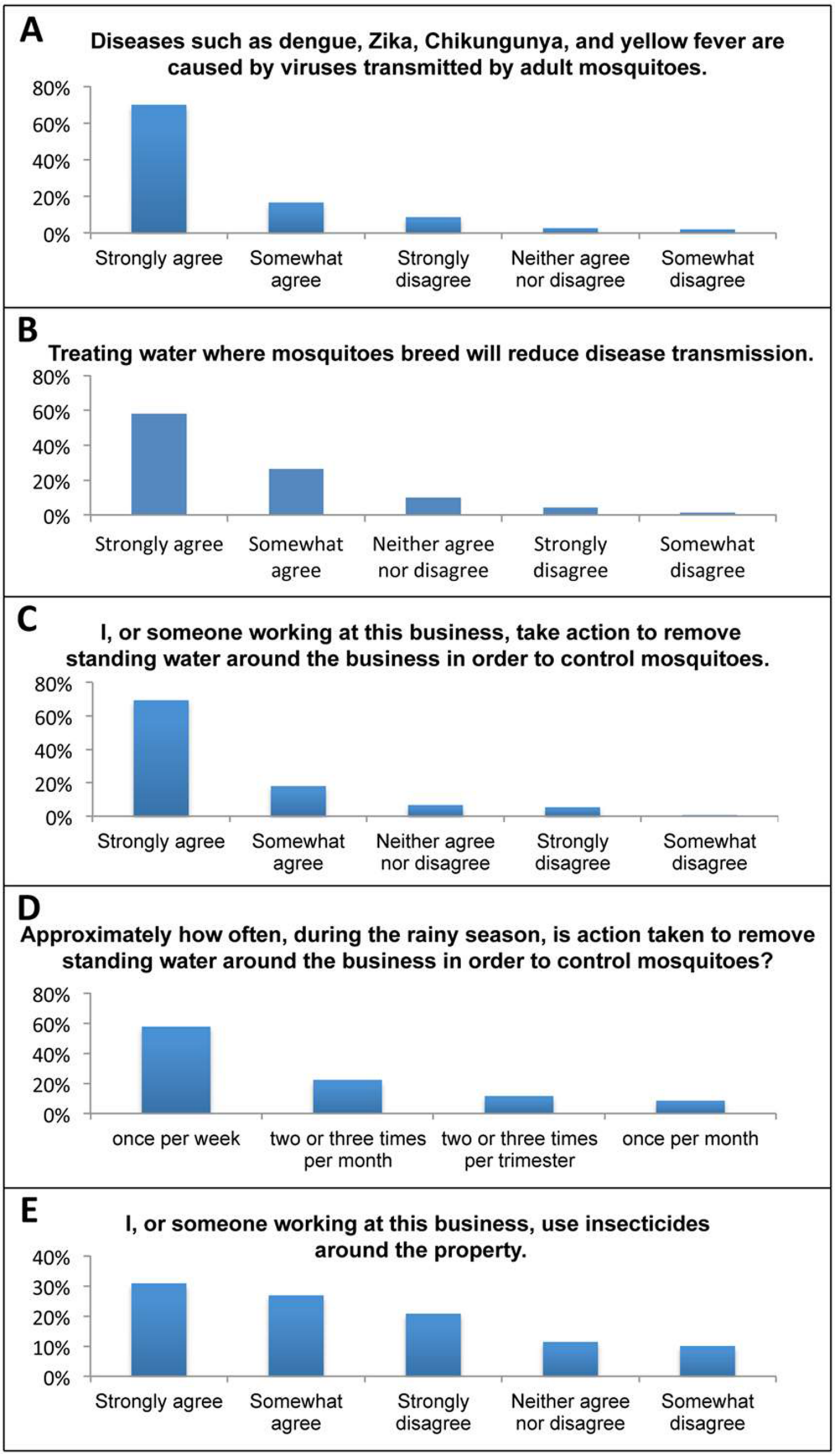
Responses to mosquito knowledge and mosquito control questions. The respondents were queried regarding their general knowledge of mosquitoes and typical mosquito control practices on their properties. Likert-scale responses concerning their agreement with the following are included: A) Mosquitoes transmit disease-causing viruses; B) Treating water where mosquitoes breed reduces disease transmission; C) Removal of standing water on the premises; D) Frequency of water removal; E) Use of insecticides on the premises. Percentages in A-E correspond to the percentage of the total respondents that provided the indicated answer (respondent count numbers are provided in Table 3).

Respondents were first provided with a set of questions that assessed their basic knowledge of mosquitoes/mosquito-borne illnesses and which examined mosquito control practices on their properties. Given the prevalence of mosquito-borne illnesses in Belize [1, 5], it was hypothesized that the survey respondents would have a reasonably good knowledge of mosquito biology and disease-causing pathogen transmission, and that most of the businesses surveyed would take some actions to reduce mosquitoes on their properties. A total of 86% of the respondents agreed (defined in this study as individuals who checked either somewhat agree or strongly agree) that mosquitoes transmit viruses that cause dengue, Zika, chikungunya, and yellow fever (Fig 3A and Table 3). Likewise, 84% of the respondents agreed that treating water where mosquitoes breed will reduce disease transmission (Fig 3B and Table 3). 87% of the respondents said that they or someone at their business regularly took action to remove standing water around the property to control mosquitoes (Fig 3C and Table 3), and 88% of these individuals indicated that they did so at least once a month or more during the rainy season (Fig 3D and Table 3). 58% of the survey respondents indicated that they used insecticides around the property (Fig 3E and Table 3). Combined, these results suggest that most economic stakeholders surveyed had reasonable knowledge of disease vector mosquitoes and made some efforts, either through larval source reduction or use of insecticides, to control mosquitoes on their properties.

**Table 3:** Mosquito knowledge and control practices.

Question	Answer	% of Responses	Count
**Diseases such as dengue, Zika, chikungunya, and yellow fever**	Strongly disagree	9%	13
**are caused by viruses transmitted by adult mosquitoes?**	Somewhat disagree	2%	3
	Neither agree nor disagree	3%	4
	Somewhat agree	17%	25
	Strongly agree	70%	105
	Total	100%	150
**Treating water where mosquitoes breed will reduce disease**	Strongly disagree	4%	6
**transmission.**	Somewhat disagree	1%	2
	Neither agree nor disagree	10%	15
	Somewhat agree	26%	39
	Strongly agree	58%	86
	Total	100%	148
**I, or someone working at this business, take action to remove**	Strongly disagree	5%	8
**standing water around the business in order to control**	Somewhat disagree	1%	1
**mosquitoes.**	Neither agree nor disagree	7%	10
	Somewhat agree	18%	27
	Strongly agree	69%	104
	Total	100%	150
**Approximately how often, during the rainy season, is action**	Once per week	58%	75
**taken to remove standing water around the business in order**	Two or three times per month	22%	29
**to control mosquitoes?**	Once per month	8%	11
	Two or three times per trimester	12%	15
	Total	100%	130
**I or someone working at this business, use insecticides**	Strongly disagree	21%	31
**around the property**	Somewhat disagree	10%	15
	Neither agree nor disagree	11%	17
	Somewhat agree	27%	40
	Strongly agree	31%	46
	Total	100%	149
**The insecticide is used to control**	Mosquitoes	75%	64
	Ants	65%	56
	Roaches	48%	41
	Bees, wasps, or hornets	11%	9
	Other	30%	26
	Total		86

Survey questions and the percentages/counts of respondents with the indicated answers are shown.

### Use of insecticides

**Fig 4:**
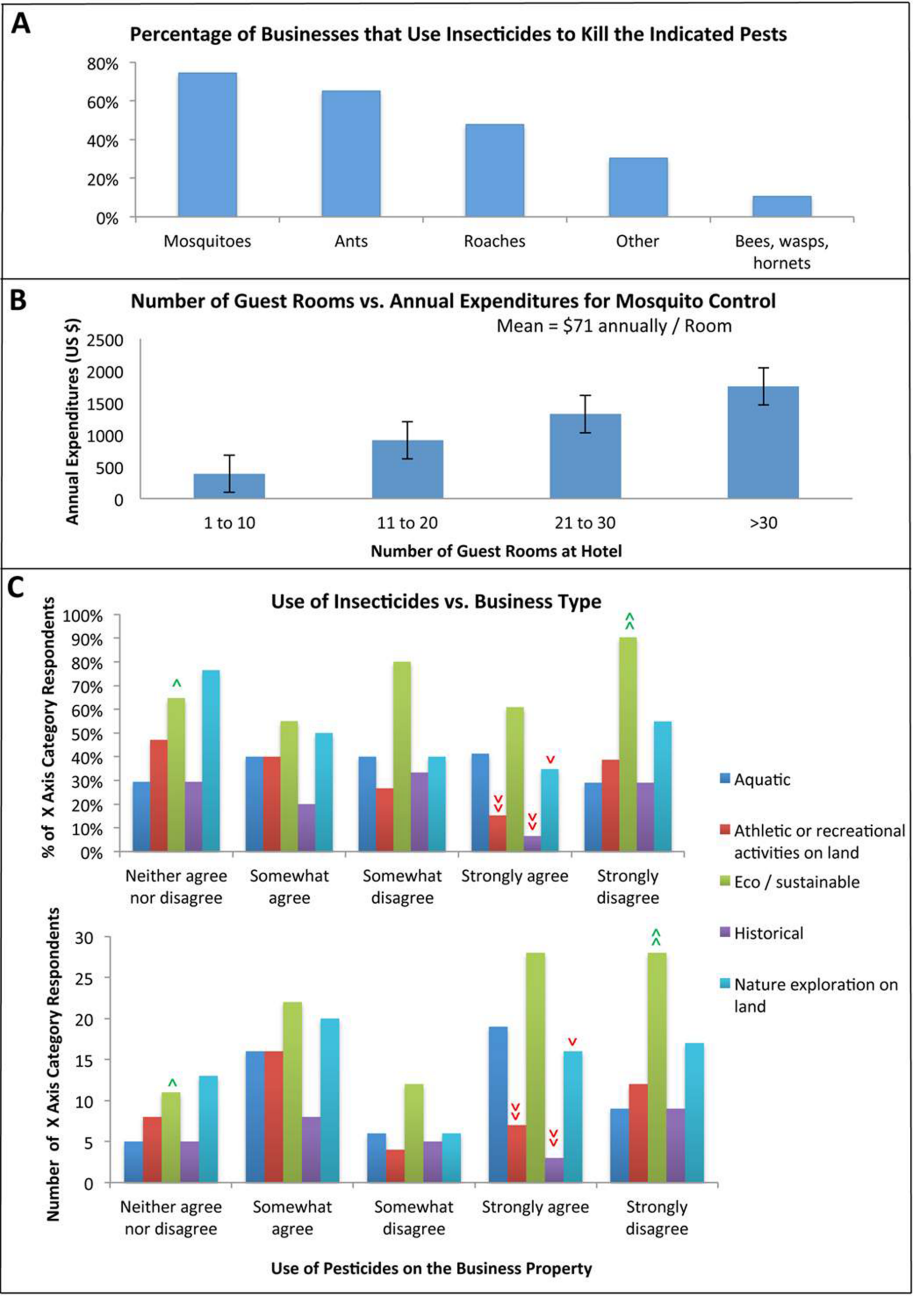
Purchase and use of insecticides. A) The percentage of insecticide-using businesses that use insecticides to kill the indicated pests. Count numbers are reported in Table 4. B) Survey-taker reported data on annual expenditures for mosquito control (converted to U.S. dollars) plotted as a function of the number of guest rooms available for rent. Annual expenditures increase as the number of rooms increases (p<0.00001). Mean = $71 spent on mosquito control per room annually. C) Likert-scale responses from representatives of the indicated types of businesses regarding the use of insecticides on their properties. The percentages (graph at top) and numbers (lower graph) of the X-axis category respondents providing the answers indicated are shown. >>> = very highly significant, >> = very significant, > = significant, with green corresponding to a higher than expected percentage/number of respondents for a given X-axis category and red corresponding to a lower than expected percentage/number of respondents for a given X-axis category. See text for additional details regarding statistical analyses and a discussion of significant results.

The survey probed more deeply into the use of insecticides at Belize tourist-related businesses. Given the substantial mosquito control program operated by the Belize MoH, it was hypothesized that smaller tourist-based businesses may not spend their own additional private funds for mosquito control on their properties. However, of the 58% of respondents who agreed that they use insecticides on the property (Table 3 and Fig 3E), 75% of these businesses indicated that the insecticides were used to control mosquitoes (Table 3 and Fig 4A). Respondents were asked to indicate how much money was spent on mosquito control by their business annually. The average among the 64 establishments that responded was $559±118/year (results were converted to U.S. dollars). The amount spent per guest room was $71/year (Fig 4B). Regression analysis indicated that there was a significant positive correlation between the amount of money spent annually and the number of guest rooms (p<0.00001), and that the amount spent annually increased by $28.70 per additional guest room (Fig 4B). Thus, the amount of money spent annually for mosquito control was dependent on the size of the hotel.

A wide variety of mosquito control products were reported to be used on the business properties (Table 4). 32% of the businesses used a liquid formulation sprayed or fogged in the air (Table 4); this was the most commonly used formulation for mosquito control, with 91% (58 of 64) of the businesses using an insecticide for mosquito control selecting this formulation. Likewise, while a variety of products were used, malathion, the chemical pesticide most commonly noted in the survey responses, was used on 30% of the properties and 20 of the 62 properties (32%) using an insecticide for mosquito control. Resistance to malathion has been observed in *Anopheles albimanus* strains in Belize, though it has not yet been assessed in Belize strains of *Aedes*. Given the high incidence of its use noted in the survey responses, in addition to its widespread use in the Belize agriculture sector, the potential for widespread resistance is of concern [21].

**Table 4:** Insecticides: Means and economics of control.

Question	Answer	% of Responses	Count
**The formulation of the product(s) used to**	Liquid sprayed or fogged in air	32%	58
**control mosquitoes around the business is**	Liquid poured into water	3%	6
**(select all that apply)?**	Solid deposited in water	4%	7
	Mosquito coil	19%	34
	Bug zapper	4%	8
	Plug-in	2%	3
	Citronella or other plant-based substance	11%	20
	Fan or air curtain	10%	19
	Screen or fabric	15%	27
	Total	100%	182
**If you know the specific name(s) of the product(s)**	Malathion	30%	20
**used around the business, please type it.**	Baygon	3%	2
	Bop	3%	2
	Fish	3%	2
	Mosquito coil	3%	2
	Shelltox	3%	2
	Argomil	1.5%	1
	Bayticol	1.5%	1
	Bifen	1.5%	1
	Cedarcide	1.5%	1
	Cyonara	1.5%	1
	Diazinon	1.5%	1
	Lorsban	1.5%	1
	Mosquito dunks	1.5%	1
	Off	1.5%	1
	Ortho Home Defense	1.5%	1
	Raid	1.5%	1
	Unknown	39%	26
	Total	100%	67
**The amount of money the business spends on**	Mean = $559 (U.S. dollars)		64
**mosquito control is ________ (currency amount)**			
**per year.**			

Survey questions and the percentages/counts of respondents with the indicated answers are shown.

It was hypothesized that businesses might opt out of insecticide use as a result of a desire to protect the environment. In support of this hypothesis, a disproportionately high percentage of the survey respondents who strongly disagreed that they would use insecticides on their properties (90%, 28 of 31) represented eco/sustainable businesses (Fig 4C, p<0.05, Χ^2^ = 12.3, d.f. = 4). Likewise, respondents representing businesses associated with athletic or recreational activities on land were significantly less likely to strongly agree to insecticide use on their properties (Fig 4B, 15% or 7 of the 46 users; p<0.05, Χ^2^ = 9.80, d.f. = 4). Businesses associated with nature exploration on land were significantly less likely to use pesticides, with only 22% (16 of 72) indicating that they strongly agreed with using insecticides for mosquito control on their properties (Fig 4B, p<0.05, Χ^2^ = 9.76, d.f. = 4). Belize is a haven for ecotourism and exploration, and ecotourism is one of the country’s largest industries. More than 27% of land in Belize is protected, and the preservation of rich biodiversity is a critical component of the ecotourism industry. This emphasis on ecotourism has unintentionally led to environmental degradation, and there is a strong movement to reverse this degradation [22]. Thus, it is perhaps not surprising that many eco/sustainable and ecotourist associated businesses are opposed to pesticide use in Belize.

### Larviciding: Knowledge and practice

The survey included a series of questions regarding the respondents’ knowledge and use of larviciding, a crucial component of integrated *Aedes* control and disease prevention programs [14]. 74% of those surveyed agreed that larvicides will reduce the number of mosquitoes (Table 5), and 85% agreed that treating water where mosquitoes breed will reduce disease transmission (Fig 3B). Despite these responses, only 31% agreed that they would plan to use larvicides on the premises in the next year (Table 5). A possible explanation for these findings is that the Belize MoH regularly employs larviciding for vector control [12], and perhaps the businesses did not find the need to pursue further larviciding. Furthermore, at least one respondent indicated in the open-ended response questions that he/she did not know where to find larvicides, which suggests that they may not be readily available throughout the country.

**Table 5:** Larviciding knowledge and practices.

Question	Answer	% of Responses	Count
**Use of larvicides will help to reduce the number**	Strongly disagree	4%	6
**of mosquitoes.**	Somewhat disagree	1%	2
	Neither agree nor disagree	21%	31
	Somewhat agree	22%	33
	Strongly agree	51%	76
	Total	100%	148
**I, or someone working at this business, intend**	Strongly disagree	29%	43
**to use larvicides to treat water on the premises**	Somewhat disagree	8%	12
**in the next year.**	Neither agree nor disagree	32%	47
	Somewhat agree	14%	21
	Strongly agree	17%	25
	Total	100%	148
**Our business would use larvicides to treat**	Decoration (vases, small ponds, or water features)	32%	59
**water intended for (select all that apply)**	Plant watering	20%	37
	Drinking, cooking, and/or bathing	7%	13
	Storage in drums or cisterns (for purposes other than drinking, cooking, or bathing)	42%	78
	Total	100%	187

Survey questions and the percentages/counts of respondents with the indicated answers are shown.

**Fig 5:**
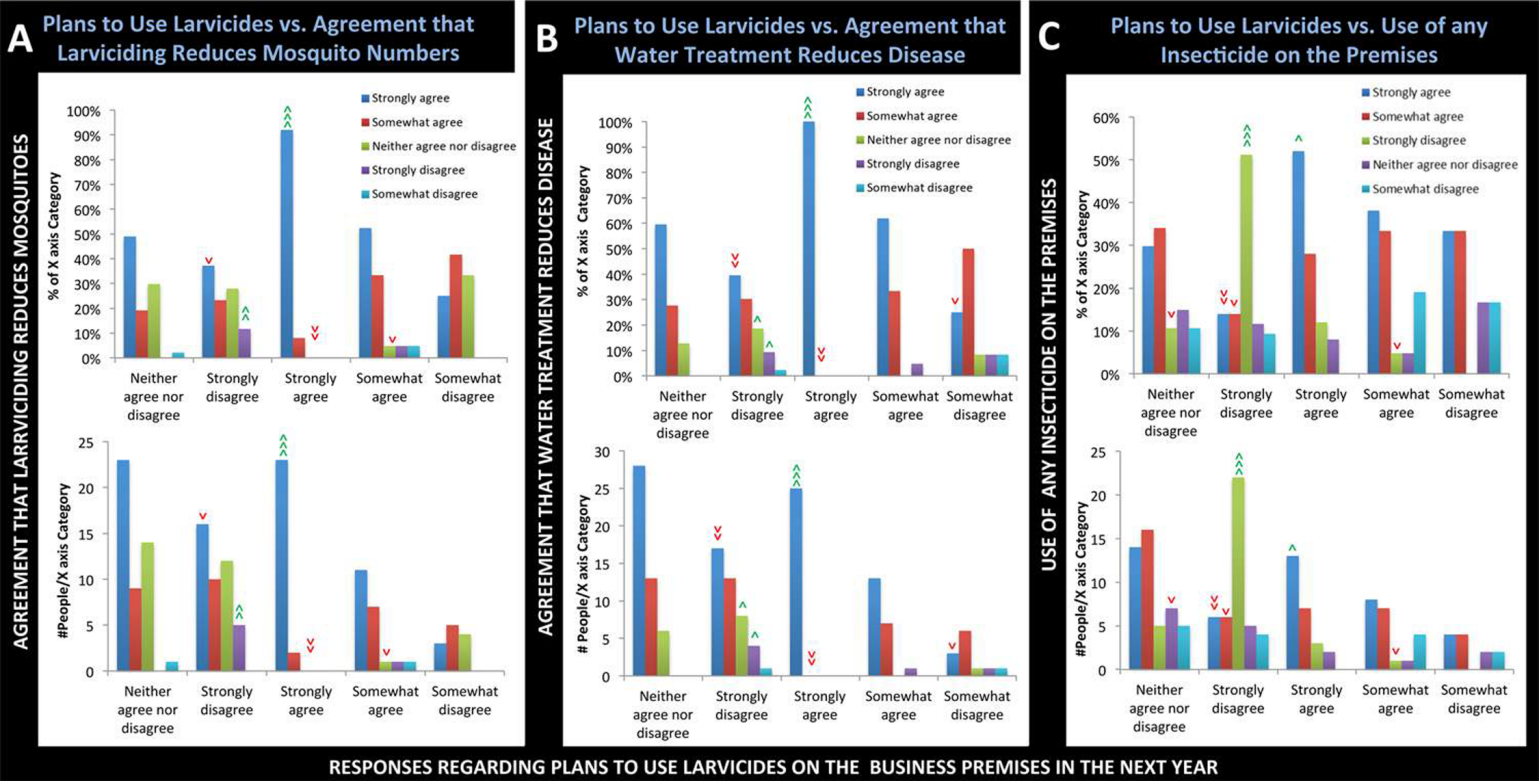
Assessment of respondents’ willingness to use larvicides on the premises. Likert-scale responses to the question of whether the business plans to use larvicides on the premises in the next year (X-axes) vs. A) Agreement that larvicide use will reduce the number of mosquitoes, B) Agreement that treatment of water where mosquitoes breed will reduce disease, and C) Respondents’ willingness to use any insecticide on the premises (Y-axes). The percentages (upper graph in each panel) and numbers (lower graphs) of the X-axis category respondents providing the answers indicated are shown. >>> = very highly significant, >> = very significant, > = significant, with green signifying a higher than expected percentage/number for a given X-axis category and red denoting a lower than expected percentage/number of respondents for a given X-axis category. See text for additional details regarding statistical analyses and discussion of significant results.

The data were further evaluated in an effort to gain a better understanding of the factors that influence the use of larvicides. It was hypothesized that larvicide use would correlate with the belief that it would reduce mosquito numbers and prevent mosquito-borne illnesses. 92% (23 of 25) of respondents who strongly agreed with larvicide use also strongly agreed that use of larvicides would reduce the number of mosquitoes (Fig 5A, p<0.001, Χ^2^ = 41.2, d.f. = 16), and 100% (25 of 25) of these individuals strongly agreed that treating water where mosquitoes breed would reduce disease transmission (Fig 5B, p<0.001, Χ^2^ = 42.6, d.f. = 16). Thus, significantly higher proportions of individuals who used larvicides on the premises believed that they would reduce the number of mosquitoes and the amount of disease transmission. Of those 46 individuals who agreed they would use larvicides on the premises, 42% indicated that they would use the larvicides to treat water stored in drums and used for purposes other than drinking, cooking, or bathing (Table 5); this was the most commonly selected use of larvicides among the positive respondents. A study in Trinidad and Tobago indicated that such larger containers were the most productive *Aedes* breeding sites [23]. Primary containers for *Aedes* breeding are yet to be fully characterized in Belize; however, drums are typical larval sources reported during routine surveillance activities. If drums are indeed the primary breeding sites, then the survey results suggest that the businesses using larvicides are focusing on treatment of the most productive containers.

Conversely, significant reductions in the strong agreement that water treatment would reduce the number of mosquitoes (16 of 43 or 37%, Fig 5A, p<0.0001, Χ^2^ = 41.2, d.f. = 16) or disease transmission (17 of 43 or 40%, Fig 5B, p<0.001, Χ^2^ = 42.6, d.f. = 16) was observed among the responders who strongly disagreed with larvicide use. Moreover, a significantly high proportion of individuals (83%; 5 of 6) who strongly disagreed that use of larvicides would reduce the number of mosquitoes also strongly disagreed that they would use larvicides on their property (p<0.001, Χ^2^ = 41.2, d.f. = 16). A significantly higher than expected proportion of individuals (67% or 4 of 6) who felt that water treatment would not reduce the number of mosquitoes also strongly disagreed that treating water where mosquitoes breed would reduce disease transmission (p< 0.00001, Χ^2^ = 181, d.f. = 16). Thus, reluctance to use larvicides strongly correlated with a lack of belief that larviciding would reduce the number of mosquitoes and disease transmission (Fig 5). These statistics reveal opportunities for educational outreach regarding the benefits of larviciding, as well as open discussion of its challenges. In support of this, the open-ended responses revealed a vast range of knowledge of larviciding, with one respondent indicating he/she didn’t know much about it and would like to learn more, while another discussed the challenges of treating cryptic breeding sites.

Finally, the decision to use larvicides correlated significantly with a willingness to use insecticides (Fig 5C). A significantly higher than expected proportion of individuals who strongly agreed with larvicide use also strongly agreed that they would use insecticides on their properties (Fig 5C, 52% or 13 of 25, p<0.0001, Χ^2^ = 46, d.f. = 16). A disproportionately high number of individuals (22 of 31 or 71%) who strongly disagreed with insecticide use also strongly disagreed that they intended to use larvicides on the premises (p<0.0001, Χ^2^ = 46, d.f. = 16). These statistics suggest that some respondents were not specifically opposed to the use of larvicides, but were more generally opposed to the use of any insecticide on their properties.

### Willingness to consider novel mosquito control technologies

The responders were queried regarding their willingness to use new products and technologies for mosquito control. 53% of the respondents agreed that they would be interested in buying a new larvicide to be used on the business premises once it had been shown to be safe and effective (Table 6). When businesses wished to consider switching to a new control product, product labels and word of mouth were the primary inputs evaluated (Table 6). Of those surveyed who strongly agreed that they would be interested in a new product, 43% (16 of 37, a significantly higher proportion than expected) also indicated that they would consider the input of a salesperson or industry representative (p<0.05, Χ^2^ = 13, d.f. = 4), and a disproportionately high percentage of those who somewhat agreed (47% or 16 of 34) indicated that their purchase choices are influenced by social media (p<0.05, Χ^2^ = 12.9, d.f. = 4).

**Table 6:** Feelings concerning novel mosquito control products and technologies.

Question	Answer	% of Responses	Count
Our business would be interested in buying a	Strongly disagree	16%	22
new type of larvicide to control mosquitoes	Somewhat disagree	8%	11
on the premises, once it has been shown to be	Neither agree nor disagree	23%	32
safe and effective.	Somewhat agree	26%	35
	Strongly agree	27%	37
	Total	100%	137
If genetically modified organisms (GMOs) were	Strongly disagree	34%	47
known to be safe and effective larvicides, this	Somewhat disagree	4%	6
business would be willing to use them.	Neither agree nor disagree	23%	32
	Somewhat agree	21%	29
	Strongly agree	18%	25
	Total	100%	139
Different forms of life can be genetically	Bacteria	40%	31
modified. Which ones would the business	Yeast	24%	19
be willing to use? (select all that apply).	Algae	36%	28
	Total	100%	78
When this business considers switching to a	Store Displays	6%	20
new product, it considers the following	Product labels	24%	78
information (select all that apply).	Radio or television advertising	4%	14
	Internet advertising	12%	40
	Social media	11%	35
	Trade show or conference	6%	19
	Salesperson or industry representative	12%	38
	Word of mouth	24%	79
	Total	100%	323

Survey questions and the percentages/counts of respondents with the indicated answers are shown.

**Fig 6:**
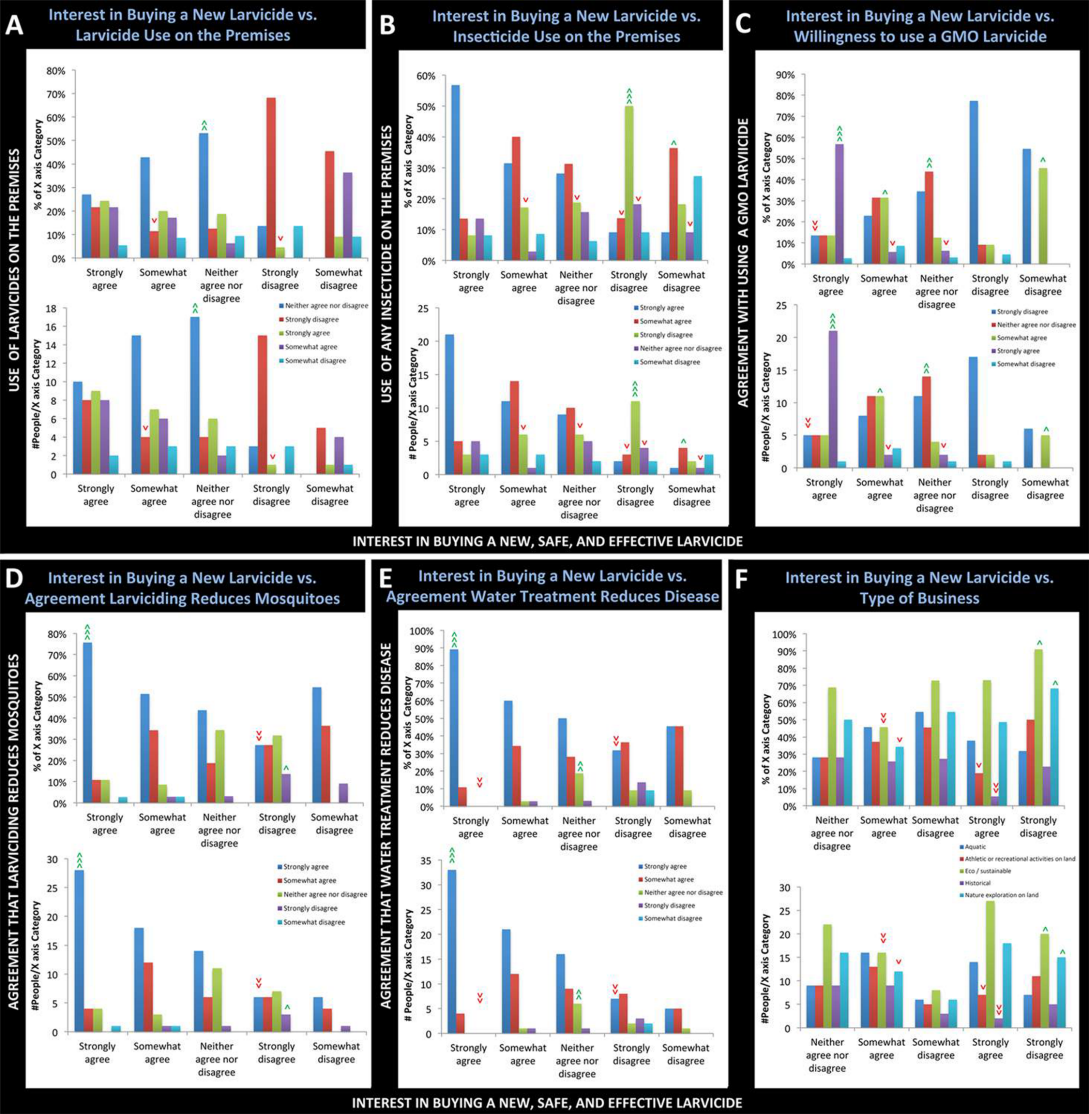
Analysis of respondents willing or not willing to purchase a new larvicide for use on the premises. Likert-scale responses to the question of whether the business is interested in purchasing a new larvicide for use on the premises (X-axes) vs. A) Willingness to use larvicides on the premises, B) Willingness to use any insecticides on the premises, C) Willingness to use a GMO-larvicide if it is shown to be safe and effective, D) Agreement that larvicide use will reduce the number of mosquitoes, E) Agreement that treatment of water where mosquitoes breed will reduce disease, and F) Respondent-provided description of the type of business. The percentages (upper graph in each panel) and numbers (lower graphs) of the X-axis category respondents providing the indicated answers are shown. >>> = very highly significant, >> = very significant, > = significant, with green representing a higher than expected percentage/number for a given X-axis category and red representing a lower than expected percentage/number of respondents for the indicated X-axis category. Additional details regarding the statistical analyses and further discussion of significant results are provided in the text.

Individuals who disagreed with the purchase of a new larvicide product at their businesses were further assessed. Of the responders who strongly disagreed with their interest in purchasing a new larvicide product, 68% (15 of 22), a disproportionately high number, also indicated that they would strongly disagree with using any larvicide on the premises in the next year (Fig 6A, p<0.0001, Χ^2^ = 47.8, d.f. = 16), while 50% (11 of 22, a disproportionately high number) strongly disagreed that any insecticide would be used (Fig 6B, p<0.001, Χ^2^ = 39.7, d.f. = 16). Of those individuals that strongly disagreed that they would be interested in purchasing a new larvicide, a significantly lower than expected percentage (27%, 6 of 22) also strongly agreed that larvicide use would reduce the number of mosquitoes (Fig 6D, p<0.01, Χ^2^ = 32.7, p<0.01), and only 32% (7 of 22), a significantly lower percentage, also strongly agreed that it would reduce disease transmission (Fig 6E, p<0.001, Χ^2^ = 43.2, d.f. = 16). Thus, a lack of interest in a new larvicide product correlated with a lack of willingness to use larvicides or insecticides in general and a lack of agreement that larviciding will reduce the number of mosquitoes or disease transmission. Moreover, of those business representatives that strongly disagreed with purchasing a new larvicide, 91% (20 of 22) were associated with eco/sustainable businesses (p<0.01, Χ^2^ = 13.8, d.f. = 4), a disproportionately high percentage (50% or 11 of 22) strongly disagreed with the use of any insecticides on their properties (Fig 6B, p<0.001, Χ^2^ = 39.7, d.f. = 16), and 68% (15 of 22, also higher than expected) also strongly disagreed that they would use larvicides on the premises (Fig 6A, p< 0.0001, Χ^2^ = 47.8, d.f. = 16). Therefore, a lack of interest in a new larvicide correlated significantly with a general reluctance to use insecticides, which was significantly linked to whether the business characterized itself as eco/sustainable (see above). The impact of mosquito control on the environment was a topic mentioned frequently in the open-ended responses and will be discussed further below.

Recent advances in the use of transgenic release strategies for vector control have highlighted the critical importance of effective community engagement prior to the use of new mosquito control technologies, particularly when genetically modified organisms (GMOs) are being considered [18, 24–27]. Others and we have proposed the use of larvicidal genetically modified microbes that express interfering RNA [16, 17, 28, 29] and recognize the importance of engaging the communities in which such interventions could potentially be used. To this end, the survey of economic stakeholders included two questions regarding the potential use of GMO larvicides, assuming that they were demonstrated to be both safe and effective.

Of those who strongly agreed to consider a new larvicide product, a significantly higher than expected number (21 of 37 or 57%) indicated that they were also willing to use a GMO as a larvicide if it were deemed safe and effective (Fig 6C, p<0.00001, Χ^2^ = 84.7, d.f. = 16). 84% (21 of 25) of those who strongly agreed with the use of GMO larvicides also strongly agreed that larviciding could reduce the number of mosquitoes (a higher percentage than expected, p<0.05, Χ^2^ = 31.1, d.f. = 16), and 88% (22 of 25) of these business representatives strongly agreed that treating water could reduce disease transmission (Fig 6E, p<0.05, Χ^2^ = 31.9, d.f. = 16). Of the respondents willing to try a GMO larvicide, they were most willing to use genetically modified bacteria (60%, Table 6), a technique which has demonstrated promise in laboratory studies [16, 17, 29]. 54% expressed willingness to use genetically modified algae (Table 6), which has also been tested in laboratory studies [30]. Finally, 37% were willing to use genetically modified yeast larvicides (Table 6), which were recently shown to generate up to 100% larval lethality in laboratory assays [16, 17].

77% (17 of 22) of those who strongly disagreed with purchasing a new larvicide product also strongly disagreed with using a GMO larvicide (Fig 6C, p<0.00001, Χ^2^ = 84.7, d.f. = 16). A very significantly high proportion (41 of 47 or 87%) of those who strongly disagreed with the use of GMO larvicides represented eco/sustainable businesses (p<0.01, Χ^2^ = 17.8, d.f. = 4), but as discussed above, 90% of these individuals were also significantly opposed to using any pesticides on the premises. Thus, their opposition to using GMO larvicides may not be specific to GMO use, but rather to the use of insecticides in general. Furthermore, only 8.7% of women (6 of 69) strongly agreed with the use of GMO larvicides, while a significantly greater than expected proportion of men (27%, 16 of 60) strongly agreed that they would be willing to use a GMO larvicide (p<0.05, Χ^2^ = 9.94, d.f. = 4). Sex-specific differences in willingness to use GMOs have been observed in other realms. For example, women have been shown to be less accepting of genetically modified foods than men [31–34]. While some have reported that years of education [31, 34, 35] and age [31] impact GMO food acceptance, neither of these variables significantly impacted GMO larvicide acceptance in this investigation.

It should be noted that although the survey respondents were queried regarding their willingness to use GMO larvicides that were demonstrated to be safe and effective, they were not provided with any details regarding the GMO larvicides that have been developed to target *Aedes* in recent years. Importantly, these larvicides have been designed so that the target sequences of the interfering RNA molecules are specific to mosquitoes and lack target sites in other organisms [16, 17]. These larvicides may therefore be safer than many chemical pesticides, but such information was not communicated to respondents in the context of this survey, which sought only to gain baseline knowledge on feelings towards larviciding and GMO larvicides in Belize. Furthermore, several studies have demonstrated that bacteria and yeast that have been genetically modified to express larvicidal interfering RNA are still effective if the microbes are first heat-killed [16, 17, 29]. Thus, the GMO larvicides under consideration are actually dead, not live GMOs, which could impact user acceptance of this intervention. Moreover, as detailed by Hapairai et al. [17], interfering RNA expression cassettes can be inserted into the yeast genome, thereby eliminating the potential for horizontal transfer or the need to use plasmids with antibiotic resistance markers, which could also influence user acceptance. In a recent community engagement study in the Florida Keys [27], when transgenic mosquito releases were under consideration, support was more commonly reported among those aware of the release, whereas those who were neutral expressed a desire for more information. Those opposed often expressed concern for the unintended consequences for disturbing natural ecosystems, while supporters often felt that such releases represented a more natural means of controlling mosquitoes than the use of insecticides. It is likely that the use of GMO larvicides may incite similar discussions. Thus, there will be many opportunities for further education and debate about the use of GMO larvicides.

### Product features that influence the purchase of mosquito control products

To gain further insight regarding current mosquito control practices and the development of new control technologies, the survey respondents were prompted to provide an open-ended response to the question *“When considering different mosquito control products for purchase*, *which product features are most important*?*”* The resulting text from 127 responses was assessed to identify words that occurred most frequently, and these results are summarized in Table 7. At the top of the list, 64 words related to safety/danger/chemical use were mentioned, with specific use of the word safe(ty) 47 times, making it the most common word found among all the responses. In total, 48% of the respondents (61 of 127) commented on product safety. Words related to environmental concerns were used 47 times, with specific mention of the word environment (or one of its derivatives) occurring 23 times. Different types of animals or plants were named 29 times. In total, 41% of respondents (52 of 127) noted concerns for the environment, animals, or plants. Furthermore, the word effective or one of its derivatives occurred 32 times, and in total, 33% of respondents (42 of 127) mentioned product efficacy as a predominant driver of product selection. These analyses suggest that safety of the products, for humans, animals, and plants, as well as general concerns for the environment, followed by product effectiveness, are the most critical determinants of product purchases. Such concerns outweighed factors such as product cost (noted by 12% of respondents; 15 of 127), lasting activity (mentioned by 5% of respondents; 6 of 127), odor (4% of responses; 5 of 127), and ease of use (3% of responses; 4 of 127). In addition to a summary of these data, quotes that effectively represent each category of responses are included in Table 7.

**Table 7:** Product features that influence the decision to purchase mosquito control products.

Theme	Occurrence	Common Words	Count	Representative Quotes
**1. Product Safety**	61 of 127 (48%)	Safety/safe	47	"Safe for humans and my animals."
		Chemical	5	"Safe for inexperienced handlers of the (chemical) product.”
**2. Environmental Impact**	52 of 127 (41%)	Environment	14	“We try to use products that are a natural base with as little environmental and human impact as possible.”
		Environmental(ly)	9	
		(Eco) Friendly	13	
		Animal(s)	8	"Non harmful to bees or other important creatures."
		Natural	5	
**3. Efficacy**	42 of 127 (33%)	Effective(ness)	32	"Effectiveness and waterproof for longevity."
				"Kills most insects that cause personal injury."

Analyses of the product features open-response question, including groups of related words and their frequencies, common words in the group and the number of times they were repeated, as well as representative quotes for each theme are shown.

It is striking that environmental concerns outweighed concerns for efficacy and cost. It is likely that the large focus on eco/sustainable tourism in Belize is a major influence on these responses. Ceballos-Lascurain [36] defined ecotourism as *“traveling to relatively undisturbed or uncontaminated natural areas with the specific objective of studying*, *admiring*, *and enjoying the scenery and its wild plants and animals*, *as well as any existing cultural manifestations (both past and present) found in these areas*.*”* As discussed by Blersch and Kangas [37], who assessed the potential for sustainability of eco-tourism in Belize, this definition has expanded to include conservation, sustainability, and ethical lines of thought [38] and embraces seven basic tenets of ecotourism: travel to natural destinations, an emphasis on environmental awareness, minimal footprints, direct financial benefits for increased conservation, the financial benefit and empowerment of local people, the respect of their culture, and support for human rights [39]. Several of these tenets, which are clearly reflected in the open-ended responses (Table 7), appear to be at the heart of tourist-based economic stakeholders’ feelings concerning acceptable means of mosquito control in Belize. As discussed below, mosquito control is undoubtedly important for the success of tourist-based businesses, but many of the stakeholders indicated that such control must have minimal impact on the environment or their health.

### Importance of mosquito control for business success

The survey respondents were asked to provide an open-ended response to the question: *“Could you please describe the importance of mosquito control for business success in Belize*?*”* Their responses were coded on a Likert-like scale, with 1 corresponding to not very important and 5 corresponding to very important. The average score (of 131 responses) was 4.17+/-0.95, corresponding to somewhat important. No significant relationships were observed in the coded response scores to this question vs. responses to any other question. A word frequency analysis was completed for both positively (score of 4 or 5) and negatively (score of 1 or 2) coded responses. Word count analyses of the negatively coded responses did not reveal any central themes, likely due to the relatively low number of negative responses (10 of 131, 8%). Further inspection of the responses indicated that most individuals with a negatively coded response simply felt that mosquito control was futile (three of 10, 30%), that mosquitoes are just part of life in Belize (three of 10, 30%), that the negative impacts on the environment outweighed the positive outcomes (two of 10, 20%), or that mosquitoes simply were not that big of an issue and are under control (two of 10, 20%).

Textual analysis of the 117 of 131 (89%) positively coded responses revealed three categories of terms observed most frequently in the responses: (1) tourists/customer-focused concerns, (2) health and disease, and (3) the environment. A summary of these analyses is presented in Table 8. Words related to tourists/customers appeared 77 times. The word tourist or one of its derivatives was included 39 times, making it the most repeated word among the responses. In total, 55 of the 117 responses (47%) noted concerns for customers. Quotes that represent the sentiment of these responses are included in Table 8. These quotes illustrate a common theme among these responses, which centered on concerns for the customers having positive experiences during their visits to the property. Next, 74 occurrences of terms related to health and disease were noted, with the word disease(s) being mentioned specifically 26 times. Responses in this category centered on the concern that mosquito control is necessary for disease prevention, and 48 of 117 responses (41%) mentioned health related items. Quotes that exemplify the nature of this category are included in Table 8. These findings are not surprising given that a majority of the respondents demonstrated reasonable knowledge of mosquito control and mosquito borne disease transmission (Table 3, Fig 3).

**Table 8:** Importance of mosquito control to tourist-based businesses.

Theme	Occurrence	Common Words	Count	Representative Quotes
**1. Tourist experience**	55 of 117 (47%)	Tourist/Tourism	39	“Our jobs depends on tourist and if the guest is getting eaten alive they will never come back to the island again.”
		Guest	23	
		Customer	5	
		Visitor/Visiting	5	"Mosquito control = tourist satisfaction."
		Travel	5	
**2. Health**	48 of 117 (41%)	Health(ier)	11	“If the country is perceived as unsafe (i.e. Zika, dengue, etc.) then our business cannot thrive.”
		Safe(ty)	11	
		Zika	8	
		Illness(es)	4	"We must protect the Belize citizens and make the tourist feel and know that steps are in place to help control the mosquito diseases.”
		Malaria	4	
**3. Environment**	26 of 117 (21%)	Environment	7	"We believe that mosquitoes can and should be controlled with eco-friendly products that do not harm the people more than the disease itself—which is what we feel about most of the products used to fight mosquitoes today."
		Chemicals	5	
		Natural	5	
		Eco	5	"Our natural treasures are what is most important to Belizeans and any mosquito control that threatens the health of humans, water systems or animals life should be avoided at all costs.”
		Toxic	4	
		Birds	4	

Analyses of the mosquito control open-ended response question, including groups of related words and their frequencies, common words in the group and the number of times they were repeated, as well as representative quotes for each theme are shown.

Finally, words related to the impact of mosquito control on the environment/unintended effects of mosquito control were mentioned 26 times. Quotes from this category (Table 8) illustrate the sentiment that mosquito control should not negatively impact the environment, a notion that was included in 21% (25 of 117) of the responses and which was reflected in several of the other data analyses summarized and discussed above.

### Economic effect of Zika vs. other mosquito-borne diseases

The respondents were given the opportunity to provide an open-ended response to the question *“Are the economic effects of Zika different than those of other mosquito-borne diseases to local businesses*?*”* Their responses were coded on a Likert-like scale, with 1 corresponding to not very different and 5 corresponding to very different. The average score among the 120 responses was 2.93±1.65 (corresponding to neither different nor not different). Once again, no significant relationships were observed in the coded scores of the responses to this question vs. responses to any other question in the survey. Next, the text from negative responses (score of 1 or 2) and positive responses (score of 4 or 5) were analyzed. 49 of 120 (41%) responses were coded negatively, while 55 of 120 (46%) were scored positively; the remaining 16 responses (13%) received a score of 3. Only 11 of the 49 negatively coded responses included a textual explanation of their response (most were just single word answers of “no” or “same”). Word count analyses of these 11 responses did not uncover specific themes. However, inspection of the 11 responses revealed several patterns. Five of 11 (45%) noted that there were few or no cases of Zika in their area or the country as a whole. 27% of respondents (3 of 11) noted that the guests had not mentioned Zika or seemed particularly concerned about it. 3 of 11 (27%) indicated that all mosquito borne illnesses were bad or that Zika was no worse than other diseases transmitted by mosquitoes. Table 9 shows the results of word counts observed in the textual analysis of the 55 positively coded responses, as well as representative quotes. Three primary themes were revealed through textual analysis of the positively coded responses: 1) concerns for the unborn and 2) strong negative feelings toward the media for their handling of the Zika crisis, both of which the respondents linked to 3) cancellations resulting from the Zika scare.

**Table 9:** The impact of Zika on tourist-based businesses in Belize.

Theme	Occurrence	Common Words	Count	Representative Quotes
**1. Concerns for Unborn**	21 of 55 (38%)	Pregnant	10	"Zika has lifelong devastating effects to pregnant or soon to be pregnant families. Not worth the risk to travel for a vacation."
		Baby/fetus	5	
		Child	4	
**2. Media**	15 of 55 (27%)	Media	11	"Media hype blew zika out of proportion."
**3. Negative impact on business**	14 of 55 (25%)	Cancellations	8	"With the negative publicity surrounding Zika we had scores of cancellations and a huge drop in business."
		Decrease/drop	3	

Analyses of the Zika impact open-ended response question, including groups of related words and their frequencies, common words in the group and the number of times they were repeated, as well as representative quotes for each theme are shown.

Congenital Zika Syndrome, a distinct pattern of birth defects that can result from fetal infection with Zika, includes several distinct features: severe microcephaly associated with partial collapse of the skull, decreased brain tissue, eye damage, congenital contractures such as clubfoot or arthrogryposis, and hypertonia that restricts body movement following birth [40]. Words related to pregnancy/babies/birth defects appeared 28 times among the positively coded responses (Table 9). 21 of 55 positive responders (38%) noted concerns for the unborn as being a primary distinguishing factor of Zika as compared to other mosquito-borne illnesses (Table 9). The results suggest that the survey responders were well informed about the potential for Zika to induce birth defects. This is quite likely due to the extensive media coverage of this topic, which was also noted in the open-ended responses. By April 2017, the time at which the survey was conducted, the number of confirmed Zika cases reported weekly was 10/week, with ~20–30 suspected cases reported during this period [5]. 17 occurrences of words related to the media were noted. Of the 55 positive responses, 15 (27%) mentioned media coverage of Zika (Table 9). The majority of these responses centered on the notion that media coverage was sensationalized, and that news coverage of Zika was a primary driver of the negative impact of Zika on tourism in Belize. 25% of the positive responses (14 of 55) noted the negative impacts on business, which the respondents directly associated with Zika and often with the extensive media coverage of it (Table 9). Words associated with cancellations and downturns in rentals were identified 15 times (Table 9). Thus, the results of the survey indicate that economic losses were incurred among the tourist-associated businesses in Belize.

At the time this survey was conducted, the United Nations Development Program issued a report [41] which concluded that the Zika epidemic would have substantial economic and social impacts, both long and short-term, in the Americas. The report noted that the impacts on countries that have tourist-based economies such as Belize and other countries in the Caribbean would be particularly strong. More than 80% of the anticipated total losses, which could reach $9 billion in the Caribbean, are the direct result of reduced revenues from international tourism. Short-term costs for Belize (2015–2017) were estimated at $35,873,714 (U.S. dollars). The report estimated that in a high infection scenario, Belize could stand to lose as much as 1.19% of GDP annually. These findings validate the concerns voiced in the survey responses. Given these significant losses, many have questioned if the media response to Zika was over-sensationalized, another clear sentiment of many of the respondents (Table 9).

Gyawali et al. [42] concluded that given the significant capacity for mosquito control in developed countries, the widespread media concern for the potential of Zika to spread to epidemic proportions in industrialized nations is difficult to justify. Samuel et al. (2018) reported that despite extensive coverage of Zika by the media, people in New York City had an overall poor understanding of Zika virus symptoms, potential complications, modes of transmission, and guidelines for prevention, and that further intervention is needed to properly educate the public. Likewise, another study noted that 40% of news articles on Zika mentioned negative potential outcomes of Zika infection without mentioning ways to prevent infections [43]. These studies suggest that, in the least, some media coverage could be improved to better educate the public about Zika prevention. Although many stakeholders in Belize felt that media coverage of Zika had a direct negative impact on their businesses (Table 9), Chandrasekaran et al. [44] reported that social media could effectively educate the public on Zika virus, concluding that young women can use social media as a useful resource on Zika. The benefits and costs of media coverage of Zika, as well as other infectious disease, will undoubtedly continue to be debated.

## Conclusions

This analysis of the results from an online survey of tourist-based business representatives from Belize revealed insight into the concerns, current mosquito control practices, and anticipated future needs of economic stakeholders working in the tourism industry in Belize. Most survey respondents demonstrated they had reasonable knowledge of mosquito disease vectors and made some efforts, either through larval source reduction or the use of insecticides, to control mosquitoes on their business properties. Use of larvicides on the business premises correlated strongly with a willingness to use insecticides in general, as well as the belief that water treatment would reduce mosquito densities and disease transmission. Over half of the respondents agreed that they would be interested in buying a new larvicide to be used on the business premises once it had been shown to be safe and effective. The safety of such products, for humans, animals, and plants, and the environment in general, followed by product effectiveness, are the most critical determinants of product purchase decisions. Although the majority of respondents agreed that control of mosquitoes and mosquito-borne illnesses was central to the success of their tourist-based businesses, many of the respondents raised concerns that the Zika epidemic had been sensationalized by the media, with dire consequences for tourist-based businesses in Belize. They also voiced concerns that current mosquito control practices, including the use of chemical pesticides, could have a negative impact on human health and the environment. Respondents, many of whom worked for eco/sustainable businesses, sought effective mosquito control interventions that have minimal impact on the environment. This study provided a framework for further engagement activities in Belize and other Caribbean nations and uncovered potential areas of concern as well as support for emerging mosquito control technologies, particularly those that are safe and eco-friendly. The results of this investigation will foster further debate and guide future educational outreach efforts in Belize and elsewhere.

## Supporting information

S1 File Economic stakeholders’ survey. The Qualtrics-formatted survey sent to tourist-based business representatives in Belize is provided as a pdf file.
(PDF)

S2 File Spanish translation of economic stakeholders’ survey. A Spanish translation of the survey is provided as a pdf file.
(PDF)
