# Supplementary material for: Mosquito control practices and perceptions: An analysis of economic stakeholders during the Zika epidemic in Belize, Central America
Source: PLoS One. 2018 Jul 19;13(7):e0201075. doi: 10.1371/journal.pone.0201075 (PMC6053204; doi:10.1371/journal.pone.0201075)
Supplement: S1 File — The Qualtrics-formatted survey sent to tourist-based business representatives in Belize is provided as a pdf file. (PDF) [file pone.0201075.s001.pdf]

English (US)

Consent

(Si desea realizar esta encuesta en Español, seleccione Español en el menú desplegable de arriba.)

You are invited to participate in a survey research study about the use of mosquito larvicides in Belize. In Belize, people can contract serious diseases through the bites of mosquitoes, and this threat has implications for the businesses of Belize, especially those whose customer base includes tourists. A large research project being conducted here will investigate new ways of preventing mosquitoes from transmitting infections. As part of this large project, we would like to learn about the perspectives of decision makers in the tourism-related Belize business sector on mosquito control.

This study is being conducted by Dr. Kathleen Eggleson of Indiana University School of Medicine-South Bend (United States), as part of a larger research project led by Dr. Molly Duman Scheel of Indiana University School of Medicine-South Bend, in collaboration with Dr. Kim Bautista of the Belize Ministry of Health and Dr. Nicole Achee & Dr. John Grieco, of The Belize Vector and Ecology Center as well as the University of Notre Dame (United States). You are invited to participate in this study because you are an adult resident of Belize who has management or executive level responsibilities in a for-profit business that operates in Belize and serves a customer base including tourists.

This study consists of a brief survey, with specific questions which will allow us to learn more about thoughts of Belize's business decision makers regarding strategies for mosquito control. As the larger research project pursues new ways of preventing mosquitoes from carrying disease, it is very important for us to consult the economic stakeholders of Belize, decision makers in businesses where products, including larvicides may be used for mosquito control. The purpose of the study is to collect information about the concerns, current practices, and anticipated future needs of mosquito control products from the perspective of the adult business leaders of Belize.

Participation in this study is voluntary and you may choose to stop participating in this study at any time.

This study consists of a brief survey, which should take about 10 minutes to complete.

If you agree to participate in this study, you will be asked to indicate your degree of disagreement or agreement, on a scale of 1 to 5, with statements pertaining to mosquito control. There will also be fill in the blank questions and questions where you can type an answer in your own words.

Participating in this study may not provide any direct benefit to you. The knowledge gained from this study will be used as part of a large research project that may result in the development of new mosquito larvicides, ultimately intended to disrupt the transmission of human disease. We do not envision any significant risks related to participation in this study.

Privacy with respect to information you will share with us if you participate in this study will be protected by the investigators. Your name and other information which would allow you to be identified as an individual are not being collected in this study, so your responses will not be attributable to you nor to the specific place of business you represent.

If you have any questions about this study, please contact Dr. Kathleen Eggleson at (574) 631-4918 (country code 1) or keggleso@nd.edu. For questions about your rights as a research participant, to discuss problems, complaints, or concerns about a research study, or to obtain information or offer input, contact the IU Human Subjects Office at 317-278-3458.

Thank you for agreeing to participate in our research. Before you begin, please note that this research is for business decision makers of Belize over the age of 18; if you are not a resident of Belize and/or under the age of 18 and/or do not work in a decision making role and/or do not work in a Belize business that serves tourists, please do not complete this survey.

The record of your informed consent will be collected by the research team, and this document will be protected as private and not shared with anyone outside of the research team. You may print this information for your records if you wish.

By clicking below, you are confirming that you: are at least 18 years of age, have read and understood the Informed Consent, and are granting your consent to participate in this study.

☐ I agree

Business

What is your professional title (for example, general manager)?

____

For what type of business do you work?

Hotel or resort	Restaurant	Tour operator	Other
☐	☐	☐	☐

Select all of the descriptions that apply to this business.

Eco / sustainable	Historical	Aquatic	Athletic or recreational activities on land	Nature exploration on land
☐	☐	☐	☐	☐

What is the star rating of this hotel or resort?

One-star	Two-star	Three-star	Four-star	Five-star	Rated, but do not know	Not rated
○	○	○	○	○	○	○

Which organization issued that star rating?

Number of rooms?

Tourists, foreign or domestic, make up what portion of the customer base of this business?

A very small part	At least half	All or almost all
○	○	○

Number of business locations in Belize?

Please mark the option which most closely represents your agreement or disagreement with the following statements.

Diseases such as dengue, Zika, chikungunya, and yellow fever are caused by viruses transmitted by adult mosquitoes (zancudos).

Strongly disagree	Somewhat disagree	Neither agree nor disagree	Somewhat agree	Strongly agree
○	○	○	○	○

Treating water where mosquitoes breed will reduce disease transmission.

Strongly disagree	Somewhat disagree	Neither agree nor disagree	Somewhat agree	Strongly agree
○	○	○	○	○

I, or someone working at this business, take action to remove standing water around the business in order to control mosquitoes.

Strongly disagree	Somewhat disagree	Neither agree nor disagree	Somewhat agree	Strongly agree
○	○	○	○	○

Approximately how often, during the rainy season, is action taken to remove standing water around the business in order to control mosquitoes?

once per week	two or three times per month	once per month	less than once per month
○	○	○	○

I, or someone working at this business, use insecticides around the property.

Strongly disagree	Somewhat disagree	Neither agree nor disagree	Somewhat agree	Strongly agree
○	○	○	○	○

The insecticide is used to control (select all that apply)

Mosquitoes/zancudos	Ants	Roaches	Bees, wasps, or hornets	Other
☐	☐	☐	☐	☐

The formulation of the product(s) used to control mosquitoes around the business is (select all that apply)

- ☐ Liquid sprayed or fogged in air
- ☐ Liquid poured into water
- ☐ Solid deposited in water
- ☐ Mosquito coil
- ☐ Bug zapper
- ☐ Plug-in
- ☐ Citronella or other plant-based substance
- ☐ Fan or air curtain
- ☐ Screen or fabric

The amount of money the business spends on mosquito control is _________ (currency amount) per year.

	Currency		Dollar Amount
	Belize	US	Please enter numbers only, without spaces, punctuation marks, or letters.
Budget for mosquito control	○	○	

If you know the specific name(s) of the product(s) used around the business, please type it/them in the box provided:

Larvicides

The following questions are specifically about larvicides (pesticides that kill mosquito larvae). Please mark the option which most closely represents your agreement or disagreement with the following statements.

Use of larvicides will help to reduce the number of mosquitoes.

Strongly disagree	Somewhat disagree	Neither agree nor disagree	Somewhat agree	Strongly agree
○	○	○	○	○

Treating water where mosquitoes breed will reduce disease transmission.

Strongly disagree	Somewhat disagree	Neither agree nor disagree	Somewhat agree	Strongly agree
○	○	○	○	○

I, or someone working at this business, intend to use larvicides to treat water on the premises in the next year.

Strongly disagree	Somewhat disagree	Neither agree nor disagree	Somewhat agree	Strongly agree
○	○	○	○	○

Our business would use larvicides to treat water intended for (select all that apply)

- ☐ Decoration (vases, small ponds, or water features)
- ☐ Plant watering
- ☐ Drinking, cooking, and/or bathing
- ☐ Storage in drums or cisterns (for purposes other than drinking, cooking, or bathing)

The following questions relate to a new type of larvicide. Please mark the option which most closely represents your agreement or disagreement with the following statements.

If genetically modified organisms (GMOs) were known to be safe and effective larvicides, this business would be willing to use them.

Strongly disagree	Somewhat disagree	Neither agree nor disagree	Somewhat agree	Strongly agree
○	○	○	○	○

Different forms of life can be genetically modified. Which ones would the business would be willing to use? (select all that apply)

- ☐ Bacteria
- ☐ Yeast
- ☐ Algae

Our business would be interested in buying a new type of larvicide to control mosquitoes on the premises, once it has been shown to be safe and effective.

Strongly disagree	Somewhat disagree	Neither agree nor disagree	Somewhat agree	Strongly agree
○	○	○	○	○

A reasonable budget for larvicides against mosquitoes is __________(currency amount) per year.

	Currency		Dollar Amount
	Belize	US	Please enter numbers only, without spaces, punctuation marks, or letters.
Budget for larvicides	○	○	

When this business considers switching to a new product, it considers the following information (select all that apply):

- ☐ Store Displays
- ☐ Product labels
- ☐ Radio or television advertising
- ☐ Internet advertising
- ☐ Social media
- ☐ Trade show or conference
- ☐ Salesperson or industry representative
- ☐ Word of mouth

Open_Questions

Could you please describe the importance of mosquito control for business success in Belize?

Are the economic effects of Zika different than those of other mosquito-borne diseases to local businesses? If yes, please describe how.

When considering different mosquito control products for purchase, which product features are most important?

Is there anything else you'd like to tell us about mosquito control for local business?

Demo

What is your gender?

Male	Female
○	○

What is your age in years?

Starting from 6 years of age, how many years of formal education have you completed?

What is your race?

Asian	American Indian / Alaska Native	Black / African American	Native Hawaiian / Other Pacific Islander	White
○	○	○	○	○

What is your ethnicity?

Not Hispanic or Latino	Hispanic or Latino
○	○

If there is another group with which you identify, please type the name below.

Powered by Qualtrics
