# Supplementary material for: Mosquito control practices and perceptions: An analysis of economic stakeholders during the Zika epidemic in Belize, Central America
Source: PLoS One. 2018 Jul 19;13(7):e0201075. doi: 10.1371/journal.pone.0201075 (PMC6053204; doi:10.1371/journal.pone.0201075)
Supplement: S2 File — A Spanish translation of the survey is provided as a pdf file. (PDF) [file pone.0201075.s002.pdf]

# Encuesta en Español

**Usted está invitado a participar en una encuesta sobre el uso de larvicidas de mosquitos/zancudos en Belice. En Belice, la gente puede contraer enfermedades graves a través de picaduras de mosquitos/zancudos, esta amenaza tiene implicaciones para los negocios de Belice, especialmente cuando sus clientes incluyen turistas. Un gran estudio que se lleva a cabo en este país investigará nuevas formas para impedir que los mosquitos/zancudos transmitan infecciones. Por eso, nos gustaría conocer las perspectivas de los tomadores de decisiones en el sector del turismo de Belice sobre el control de mosquitos/zancudos.**

**Este estudio está conducido por la Dra. Kathleen Eggleson de la Escuela de Medicina de la Universidad de Indiana-South Bend (Estados Unidos), parte de un proyecto dirigido por la Dra. Molly Duman Scheel de la Escuela de Medicina de la Universidad de Indiana-South Bend, en colaboración con el Dr. Kim Bautista del Ministerio de Salud de Belice y la Dra. Nicole Achee y el Dr. John Grieco, del Centro de Ecología y Vectores de Belice y de la Universidad de Notre Dame (Estados Unidos). Usted está invitado a participar en este estudio porque usted es un residente adulto de Belice que tiene responsabilidades de gestión o de nivel ejecutivo en un negocio con fines de lucro que opera en Belice y sirve a clientes incluyendo turistas.**

**Este estudio consiste en una breve encuesta, con preguntas específicas que nos permitirán aprender más acerca de los pensamientos de los tomadores de decisiones de las empresas beliceñas sobre las estrategias contra los mosquitos/zancudos. A medida que se añaden investigaciones sobre nuevas formas de evitar que los mosquitos/zancudos porten enfermedades, es muy importante que consulten a los actores económicos Beliceños, tomadores de decisiones de empresas donde se pueden usar productos larvicidas para controlar los mosquitos/zancudos. El propósito del estudio es recopilar información sobre las preocupaciones, las prácticas actuales y anticipar las necesidades futuras de los productos de control de mosquitos/zancudos desde la perspectiva de los líderes empresariales adultos de Belice.**

**La participación en este estudio es voluntaria y usted puede dejar de participar en este estudio en cualquier momento.**

**Este estudio consiste en una breve encuesta tomando unos 10 minutos para completar.**

**Si acepta participar en este estudio, se le pedirá que indique su grado de desacuerdo o acuerdo, en una escala de 1 a 5, al sujeto del control de mosquitos/zancudos. También habrá preguntas a completar y las preguntas en las que puede responder con sus propias palabras.**

**Participar en este estudio puede no proporcionar ningún beneficio directo para usted. El conocimiento adquirido de este estudio se utilizará como parte de un gran estudio de investigación que puede resultar en el desarrollo de nuevos larvicidas de mosquito/zancudo, con el fin de interrumpir de la transmisión de enfermedades. No anticipamos riesgos significativos con su participación en este estudio.**

**La información privada que compartirá con nosotros si participa en este estudio estará protegida por los investigadores. Su nombre y otra información que le permitiría ser identificado como individuo no está siendo encuestada en este estudio, por lo que sus respuestas no serán atribuibles a usted ni al lugar específico del negocio que usted representa.**

**Si tiene alguna pregunta sobre este estudio, comuníquese con la Dra. Kathleen Eggleson al (574) 631-4918 (código de país 1) o keggleso@nd.edu. Para preguntas acerca de sus derechos como participante de investigación, para discutir problemas, quejas o inquietudes acerca de la investigación, o para obtener información o sugerencias, comuníquese con la Oficina de Sujetos Humanos de IU al 317-278-3458.**

**Gracias por aceptar participar en nuestra investigación. Antes de comenzar, tenga en cuenta que esta investigación es para los tomadores de decisiones comerciales Beliceños mayores (+18 años); Si no es residente de Belice y / o es menor y / o no trabaja en un puesto que toma de decisiones y / o no trabaja en un negocio de Belice que atiende a turistas, por favor no complete esta encuesta.**

**El registro de su consentimiento será recopilado por el equipo de investigación, y este documento será mantenido privado y no compartido con nadie fuera del equipo de investigación. Puede imprimir esta información para sus registros si lo desea.**

**Al hacer clic abajo, usted confirma que usted: tiene al menos 18 años de edad, ha leído y entendido el Consentimiento y está otorgando su consentimiento para participar en este estudio.**

**¿Cuál es su título profesional?**

**¿Cuál es la categoría de estrellas que tiene este hotel o resort?**

| | | | | | | |
|---|---|---|---|---|---|---|
| 1- estrella | 2- estrellas | 3- estrellas | 4- estrellas | 5- estrellas | Tiene, pero no se | Sin categoría |

**¿Para qué tipo de empresa trabaja?**

| | | | |
|---|---|---|---|
| Hotel or resort | Restaurante | Operadores turísticos | Otro |

---

**Seleccione todo(s) lo(s) que se aplica(n) a esta empresa.**

| | | | | |
|---|---|---|---|---|
| Eco / sostenible | Histórico | Acuático | Atlético/ Actividades recreativas terrestre | Exploración de naturaleza terrestre |

**¿Cuál es la categoría de estrellas que tiene este hotel o resort?**

| | | | | | | |
|---|---|---|---|---|---|---|
| 1- estrella | 2- estrellas | 3- estrellas | 4- estrellas | 5- estrellas | Tiene, pero no se | Sin categoría |

**¿Número de habitaciones?**

**Los turistas, extranjeros o locales, constituyen qué parte de los clientes de esta empresa?**

| | | |
|---|---|---|
| Una pequeña parte | Al menos la mitad | Todo/mayoría |

---

**Número de establecimientos comerciales en Belice**

**Por favor seleccione la opción que más representa su nivel de acuerdo o desacuerdo con las declaraciones siguientes.**

---

**Enfermedades como el dengue, Zika, chikungunya y la fiebre amarilla son causadas por virus transmitidos por mosquitos (zancudos) adultos.**

| | | | | |
|---|---|---|---|---|
| Totalmente en desacuerdo | Parcialmente en desacuerdo | Ni de acuerdo ni en desacuerdo | Parcialmente de acuerdo | Totalmente de acuerdo |

---

**El tratamiento del agua donde se crían los mosquitos reducirá la transmisión de enfermedades.**

| | | | | |
|---|---|---|---|---|
| Totalmente en desacuerdo | Parcialmente en desacuerdo | Ni de acuerdo ni en desacuerdo | Parcialmente de acuerdo | Totalmente de acuerdo |

**Yo, o otro empleado en este empresa, tomo(a) medidas para eliminar el aqua estancada alrededor de la propiedad con el fin de controlar los mosquitos.**

| | | | | |
|---|---|---|---|---|
| Totalmente en descuerdo | Parcialmente en desacuerdo | Ni de acurerdo ni en desacuerdo | Parcialmente de acuerdo | Totalmente de acuerdo |

**Aproximadamente, ¿con qué frecuencia, durante la temporada de lluvias, se toman medidas para eliminar el agua estancada alrededor de la propiedad para controlar a los mosquitos?**

| | | | |
|---|---|---|---|
| Una vez por semana | Dos o tres veces por mes | Una vez por mes | Al menos una vez al mes |

**Yo, o otro empleado en esta empresa, uso(a) insecticidas alrededor de la propiedad.**

| | | | | |
|---|---|---|---|---|
| Totalmente en desacuerdo | Parcialmente en desacuerdo | Ni de acuerdo ni en desacuerdo | Parcialmente de acuerdo | Totalmente de acuerdo |

**El insecticida se utiliza para controlar (seleccione todo(s) lo(s) que se aplique(n))**

| | | | | |
|---|---|---|---|---|
| Mosquitos/zancudos | Hormigas | Cucarachas | Abejas, avispas, avispones | Otro |

**Las preguntas siguientes se refieren específicamente a los larvicidas (pesticidas que matan las larvas de los mosquitos). Por favor seleccione la opción que más representa su nivel de acuerdo o desacuerdo con las declaraciones siguientes.**

---

**El uso de larvicidas ayudará a reducir el número de mosquitos.**

| | | | | |
|---|---|---|---|---|
| Totalmente en desacuerdo | Parcialmente en desacuerdo | Ni de acuerdo ni en desacuerdo | Parcialmente de acuerdo | Totalmente de acuerdo |

---

**El tratamiento del agua donde se reproducen mosquitos reducirá la transmisión de enfermedades.**

| | | | | |
|---|---|---|---|---|
| Totalmente en desacuerdo | Parcialmente en descuerdo | Ni de acuerdo ni en descuerdo | Parcialmente de acuerdo | Totalmente de acuerdo |

**Yo, o otro empleado en esta empresa, pienso(a) usar larvicidas para tratar el agua en las instalaciones en el próximo año.**

| | | | | |
|---|---|---|---|---|
| Totalmente en desacuerdo | Parcialmente en desacuerdo | Ni de acuerdo ni en desacuerdo | Parcialmente de acuerdo | Totalmente de acuerdo |

**Nuestra empresa utilizaría larvicidas para tratar el agua destinada para: (seleccione todo(s) lo(s) que se aplique(n))**

- Decoración (jarrones, pequeños estanques, fuentes de agua)
- Riego de plantas
- Beber, cocinar y / o bañarse
- Almacenamiento en tambores o cisternas (para otros fines que no sean beber, cocinar o bañarse)

**Las preguntas siguientes se refieren específicamente a un nuevo tipo de larvicida. Por favor seleccione la opción que más representa su nivel de acuerdo o desacuerdo con las declaraciones siguientes.**

---

**Si se supiera que los organismos genéticamente modificados (OGM) son larvicidas seguros y eficaces, esta empresa estaría dispuesto a utilizarlos.**

| Totalmente en desacuerdo | Parcialmente en desacuerdo | Ni de acuerdo ni en desacuerdo | Parcialmente de acuerdo | Totalmente de acuerdo |
|---|---|---|---|---|

**Diferentes formas de vida pueden ser modificadas genéticamente. ¿Cuáles estaría dispuesto a utilizar en la empresa? (seleccione todo(s) lo(s) que aplique(n))**

- Bacterias
- Levadura
- Algas

**Nuestra empresa estaría interesada en comprar un nuevo tipo de larvicida para controlar mosquitos en las instalaciones, una vez que se ha demostrado que es seguro y eficaz.**

| Totalmente en desacuerdo | Parcialmente en desacuerdo | Ni de acuerdo ni en desacuerdo | Parcialmente de acuerdo | Totalmente de acuerdo |
|---|---|---|---|---|

**Un presupuesto razonable para larvicidas contra mosquitos es ________ (moneda) por año.**

| | Monedad | | Cuantos |
|---|---|---|---|
| | Belice | Estados Unidos | Solamente numeros, sin espacios, puntuación, o letras, por favor. |
| Persupuesto para larvicidas | ○ | ○ | |

**Cuando nuestra empresa considera cambiar a un nuevo producto, la empresa considera la información siguiente:**
**(seleccione todo(s) lo(s) que se aplique(n))**

| | |
|---|---|
| Cartel en tiendas | Medios de comunicación social |
| Etiquetas de producto | Ferias or congresos |
| Publicidad de radio o televisión | Vendedor o representante industrial |
| Publicidad sobre Internet | Por referencia verbal |

**¿Podría usted describir la importancia del control de mosquitos para el éxito empresarial en Belice?**

**¿Son los efectos económicos del Zika diferentes a las otras enfermedades transmitidas por mosquitos para las empresas locales? En caso afirmativo, describa cómo.**

**Cuando considera comprar diferentes productos de control de mosquitos, ¿cuáles son las características más importantes del producto?**

**¿Son los efectos económicos del Zika diferentes a las otras enfermedades transmitidas por mosquitos para las empresas locales? En caso afirmativo, describa cómo.**

**¿Hay algo más que le gustaría decirnos sobre el control de mosquitos para negocios locales?**

**Cuál es su género?**

| | |
|---|---|
| Masculino | Femenino |

**¿Cuál es su edad en años?**

**A partir de los seis años de edad, ¿cuántos años de educación formal ha completado?**

**¿Cuál es su grupo racial?**

| | | | | |
|---|---|---|---|---|
| Asiático | Nativo Americano/ Nativo Alaskan | Afroamericano | Nativo Hawaiano/ Otro Isleño Pacifico | Caucásico |

**¿Cuál es su grupo étnico?**

| | |
|---|---|
| No Hispano o Latino | Hispano o Latino |

**Si hay otro grupo con el que se identifica, escriba el nombre abajo.**

Gracias por dedicarle tiempo a esta encuesta.
Su respuesta se ha registrado.
